# Extracting Features from Poincaré Plots to Distinguish Congestive Heart Failure Patients According to NYHA Classes

**DOI:** 10.3390/bioengineering8100138

**Published:** 2021-10-03

**Authors:** Giovanni D’Addio, Leandro Donisi, Giuseppe Cesarelli, Federica Amitrano, Armando Coccia, Maria Teresa La Rovere, Carlo Ricciardi

**Affiliations:** 1 Institute of Care and Scientific Research Maugeri, 27100 Pavia, Italy; gianni.daddio@icsmaugeri.it (G.D.); giuseppe.cesarelli@unina.it (G.C.); federica.amitrano@unina.it (F.A.); armando.coccia@unina.it (A.C.); mariateresa.larovere@icsmaugeri.it (M.T.L.R.); carloricciardi.93@gmail.com (C.R.); 2 Department of Advanced Biomedical Sciences, University of Naples Federico II, 80131 Naples, Italy; 3 Department of Chemical, Material and Production Engineering, University of Naples Federico II, 80125 Naples, Italy; 4 Department of Information Technology and Electrical Engineering, University of Naples Federico II, 80125 Naples, Italy

**Keywords:** congestive heart failure, heart-rate variability, machine learning, NYHA classification, Poincaré plot analysis

## Abstract

Heart-rate variability has proved a valid tool in prognosis definition of patients with congestive heart failure (CHF). Previous research has documented Poincaré plot analysis as a valuable approach to study heart-rate variability performance among different subjects. In this paper, we explored the possibility to feed machine-learning (ML) algorithms using unconventional quantitative parameters extracted from Poincaré plots (generated from 24-h electrocardiogram recordings) to classify patients with CHF belonging to different New York Heart Association (NYHA) classes. We performed in sequence the following investigations: first, a statistical analysis was carried out on 9 morphological parameters, automatically measured from Poincaré plots. Subsequently, a feature selection through a wrapper with a 10-fold cross-validation method was performed to find the best subset of features which maximized the classification accuracy for each considered ML algorithm. Finally, patient classification was assessed through a ML analysis using AdaBoost of Decision Tree, k-Nearest Neighbors and Naive Bayes algorithms. A univariate statistical analysis proved 5 out of 9 parameters presented statistically significant differences among patients of distinct NYHA classes; similarly, a multivariate logistic regression confirmed the importance of the parameter $\rho_{y}$ in the separability between low-risk and high-risk classes. The ML analysis achieved promising results in terms of evaluation metrics (especially the Naive Bayes algorithm), with accuracies greater than 80% and Area Under the Receiver Operating Curve indices greater than 0.7 for the overall three algorithms. The study indicates the proposed features have a predictive power to discriminate the NYHA classes, to which the features seem evenly correlated. Despite the NYHA classification being subjective and easily recognized by cardiologists, the potential relevance in the clinical cardiology of the proposed features and the promising ML results implies the methodology could be a valuable approach to automatically classify CHF. Future investigations on enriched datasets may further confirm the presented evidence.

## 1. Introduction

Heart-rate variability (HRV) measurements over the years have proven to be a valuable aid in the study of cardiorespiratory control systems under various pathological conditions and, particularly, in the definition of the prognosis in patients with myocardial infarction and congestive heart failure (CHF). Indeed, Malik et al. [1] indicated that HRV plays an important role in diagnosis and treatment of cardiovascular diseases such as CHF, which is a difficult condition in clinical treatment and has a high mortality rate [2,3,4]. Physiological and clinical considerations have suggested a multiplicity of approaches to study the HRV. These consist of deterministic analyses in the time and frequency domains [5], stochastic analyses in the frequency domain and, more recently, chaotic approaches based on non-linear methods [6]. An interesting solution, since it does not consider continuous series data over time nor a normal distribution of signals samples as in the time domain or frequency analysis, is Poincaré plot. Moreover, this approach does not require an excessive computational burden and it is graphic. The Poincaré plot technique is based on the analysis of maps which illustrate each RR interval—extracted from electrocardiogram (ECG) recordings—against the previous one.

The New York Heart Association (NYHA) functional class is widely used in clinical practice for evaluating the severity of functional limitations from a patient’s heart failure condition. Classification is based on the symptoms a patient experiences during activity [7].

Machine learning (ML) has been used deeply in recent years to find hidden patterns in data or to build models for classification and prediction [8]. In healthcare, ML was successfully applied in several specialties: for instance, studies in neurology have showed ML algorithms and gait analysis might distinguish parkinsonian patients’ symptoms studying many spatial and temporal parameters [9,10]; again, several radiomics studies in oncology have used ML to identify tumor grade [11,12].

ML has been widely employed and discussed in the literature for its application in cardiology [13]: it has been used for cardiac imaging applications such as the automated computation of scores, the differentiation of prognostic phenotypes, the quantification of heart function, the segmentation of the heart and the diagnosis of coronary artery disease [14,15]. ML has also contributed to cardiovascular risk assessment and to predict cardiovascular events [16].

Previous research has documented the implementation of ML algorithms in this field. For instance, Isler and co-workers [17] studied the optimal features subsets combinations to discriminate CHF patients from healthy control subjects developing a multi-stage classification process to maximize diagnosis accuracy. These results were achieved considering different classes of features (including a few extracted from Poincaré plot), preliminary results from a one-step classification process using different ML algorithms and results variations for these algorithms comparing the effects of different cross-validation methods [18]. Gong and co-workers [19] addressed a similar objective investigating possible improvements in ML classification stages studying computational testing time. The use of a specific feature subset (3 Poincaré plot features out of 10) based on a histogram-manual feature selection—extracted from segmented 5 min ECG recordings—allowed a neural network to effectively discriminate arrhythmia and normal state signals in several (about 200) milliseconds. Zhao and co-workers [20] proposed in a recent paper the simultaneous extraction of several features from HRV and pulse transit time variability data to enhance CHF detection using a ML algorithm. The authors demonstrated an increasing classification performance. Finally, Agliari and co-workers [21] developed a multi-label classification algorithm (feed-forward neural network) discriminating healthy versus cardiac subjects. The authors fed the neural network using standard clinical markers (features) extracted from 24-h Holter recordings (1 feature from Poincaré plot). They demonstrated a classification accuracy of ~80–85% on a sample of more than 2200 real patients.

Despite the promising results presented in the last paragraph, previous ML studies have aimed at distinguishing CHF severity considering NYHA classification and proved the feasibility of HRV indexes in classifying patients according to this consolidated clinical scale [22,23,24]; nevertheless, studies based only on features extracted from Poincaré plot analysis are still lacking. Therefore, paper purpose is double. On the one hand, the objective is to feed ML algorithms using quantitative parameters extracted from Poincaré plot (as described earlier in [25]) to effectively classify patients affected by a different severity of CHF; on the other hand, the goal is to underline the predictive power of these unconventional features extracted from the Poincaré plots. These may be potentially useful in the cardiological clinical setting to manage different cardiac issues in which the study of the heart-rate variability is of paramount importance. This paper is an extension of two previous pilot studies which showed the 9 extracted features (fed to specific ML algorithms) demonstrated to discriminate different cardiac diseases [26] and distinguish patients in three NYHA classes by also using techniques for balancing the dataset with artificial data [27]. In this new study, the as—is dataset has been employed without the introduction of artificial data.

The implementation of ML-based tools in physiology, particularly in the cardiovascular area, has become more and more important; these tools have transformed the framework of biomedical research and the introduction of new parameters (i.e., extracted from Poincaré plot analysis) for performing the classification of the severity of CHF according to the NYHA could potentially support physiologists suggesting specific decisions.

## 2. Materials and Methods

### 2.1. Study Population

One hundred and ninety-nineth stable patients affected by CHF in sinus rhythm ranging from mild (NYHA I) to moderate (NYHA III) were hospitalized to the Heart Failure Unit of the Institute of Care and Scientific Research Maugeri (Italy) for assessment and therapy of the CHF. The dataset was the same used in the research of Maestri et al. having excluded only the patient with NYHA class equal to IV since only one subject could not be representative of the entire population [28]. According to the 2016 European Society of Cardiology “Acute and Chronic Heart Failure” Guidelines, this study considered only patients with reduced ejection fraction (<40%). Subjects with mid-range and preserved ejection fraction were discarded. Moreover, all the inclusion and exclusion criteria, the clinical pathway designed for patients and the instrumentation for the exams are described in the previous research [28]. Some criteria for the election of patients were related to sinus rhythm, stable clinical conditions and the absence of diseases affecting the autonomic control of cardiovascular function, including insulin-dependent diabetes, 24-h Holter recording analyzable for at least half of the night-time and half of the daytime. During the first week after the Holter recording, the selected patients received a two-dimensional echocardiography, a cardiopulmonary exercise testing and blood tests. NYHA patients belonged to classes I, II and III result 22, 116 and 61, respectively. The clinical characteristics of the population studied are listed in Table 1. All the patients enrolled gave their informed consent, the local Ethics Committee approved the study, which was performed in accordance with Declaration of Helsinki.

### 2.2. Poincaré Plot Analysis

As an alternative to the classic approaches to study HRV in the time and frequency domains, it is possible to study the beat-beat variability using Poincaré plot, the prognostic value of which has already been demonstrated in the literature for patients affected by CHF [29]. The morphology of such plots, known as Poincaré plot, Lorentz plot or scatter plot for 24-h Holter recordings was described in the literature [30], which has documented a classification in four typical patterns (Comet, Torpedo, Fan and Complex). Some works described the prognostic value of visual inspection of such plots for CHF patients, showing a correlation with the risk of mortality higher than that derived from traditional analyses in the time and frequency domains [31,32]. Some authors have tried to quantify different morphological parameters of Poincaré plot [33]: a clear correlation has been verified between the parameters in the time domain and some parameters achievable from Poincaré plot, although calculated only manually [34,35]. However, different parameters describing changes in heart signal variability can only be considered clinically reliable only if their reproducibility is demonstrable. Studies on the reproducibility of parameters in the time and frequency domains in both short and long term can be found widely in the literature [5,36], as the unconventional quantitative parameters extracted from Poincaré plot which are described in detail below [25,33,37]. ECG 24-h Holter recordings were performed with the portable 3-channel recorder Marquette 8000 T (Marquette Electronics, currently General Electric Healthcare Inc. Milwaukee, WI, USA). Subsequently, Holter recordings were processed by means of an Elatec system (software version 3.0; ElaMedical, S.p.A.). Each beat was first automatically labeled as normal or aberrant by the Holter analysis software and then carefully edited by an expert analyst. Annotated RR time series were processed to correct for isolated ectopic beats (linear interpolation), artefact and runs of ventricular or supraventricular beats. [28]. Finally, a dedicated software developed by the authors [34,38] allowed to automatically compute the main morphological features extracted from the Poincaré plot. Only normal classified QRS complexes were considered in the analysis excluding RR intervals preceding or following not-normal beats and plotting only time-closed RR couples [25].

**Figure 1:**
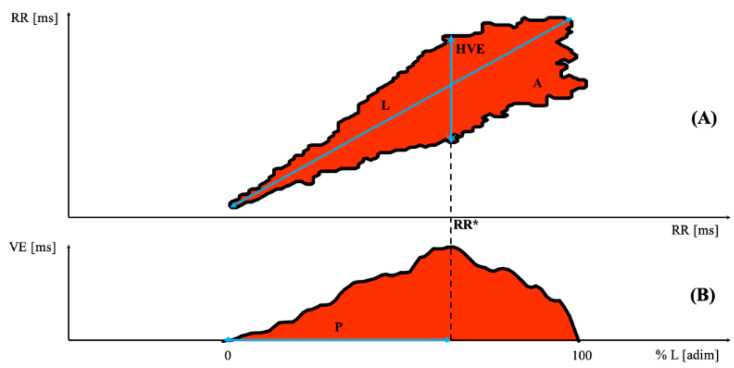
(**A**) Example of a 2D scatter plot RR vs. RR presenting the visual representation of the following extracted parameters: length (L [ms]), highest variability extension (HVE [ms])—related to the RR value showing the maximum width (RR *)—and area (A [(ms)^2^]). (**B**) Curve which represents the measure of VE at different RR intervals; the percentage of the length which corresponds to the maximum amplitude (HVE) of the plot is P ([%]).

The developed software has allowed to automatically derive the following parameters extracted from the 2D plots (see Figure 1). Briefly, the following tasks were carried out to analyze the 2D Poincaré plot. Algorithms for binary image analysis were applied on 2D Poincaré plot to eliminate salt and pepper noise (isolated points or points below a default degree of connection), the presence of which would have incorrectly altered the estimation of these parameters. To reach this objective, all connected components, namely objects that have fewer than four pixels from the binary image (namely the 2D Poincaré Plot), were removed; this operation is known as an area opening. Moreover, a flood fill operation on background pixels of the input binary image was performed, starting from the points specified.

Four features were extracted following the aforementioned operations: Highest Variability Extension (HVE [ms]), Length (L [ms]), Area (A [ms]) and the percentage of the L which corresponds to HVE (P [%]), as shown in Figure 1. The variability extension (VE) function is obtained scanning the 2D Poincaré plot (Figure 1A) with a vertical line, generating a curve which represents the measure of scatter plot width at different RR intervals (Figure 1B) [33,38]. HVE is the maximum of VE which corresponds to RR *.

**Figure 2:**
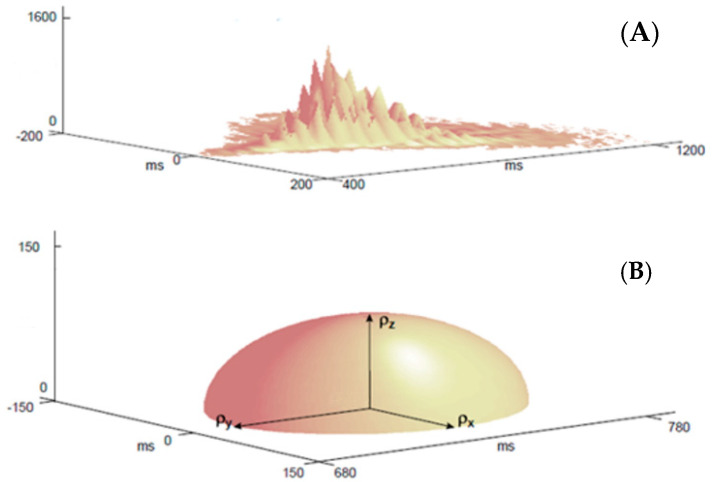
(**A**) Example of a 3D scatter plot presenting the visual representation of the following parameters: number of peaks (N_p_ [adim]), mean distance of peaks from the axis of symmetry (D_p_ [ms]). (**B**) Example of the semi-ellipsoid of inertia illustrating the coordinates of the three radii of inertia $:\rho_{x}\;\left[\mathrm{ms}\right],\rho_{y}\left[\mathrm{ms}\right],\rho_{z}\;\left[\mathrm{adim}\right]$.

The following 3D features have been extracted from the 3D plots using again the developed software: number of peaks (N_p_ [adim]), mean distance of peaks from the axis of symmetry (D_p_ [ms]), and the three inertia radii of the semi-ellipsoid of inertia ($\rho_{x}$ [ms], $\rho_{y}$ [ms], $\rho_{z}$ [adim]), as shown in Figure 2. The peaks showed in Figure 2A were identified by a threshold value defined in percent of the maximum. To select a threshold as independent as possible from the number of identified peaks, it has been observed that for the threshold value equal to half the maximum, the peak count tends to stabilize, namely it grows rapidly for lower values and much less quickly for higher values, and the choice of a threshold too low or too high would lead to overestimate or underestimate the presence of significant peaks, respectively. [33]. By looking at the 3D plot (Figure 2A), as composed of point masses of a discrete material system of N points, it is possible to collect information about their spatial dispersion in three dimensions by computing the semi-ellipsoid of inertia (considering that the points are all positive). (X_g_ [ms], Y_g_ [ms], Z_g_ [adim]) are the triplet of the barycenter of the distribution of the points and ($\rho_{x}$, $\rho_{y}$, $\rho_{z}$) is the radii of inertia of the considered surface quadric (Figure 2B).

### 2.3. Statistical Analysis

A preliminary statistical analysis was carried out to investigate the dataset. First, a univariate statistical analysis was performed for each parameter extracted from the Poincaré plot analysis. A Kolmogorov Smirnov test for normality was performed to investigate the distribution of the data with a level of uncertainty of 0.05. Then, ANOVA or a Kruskal Wallis tests were performed to distinguish the classes of NYHA. Finally, a post-hoc test was performed whether the previous tests resulted significant (*p*-value < 0.05).

Secondly, a multivariate logistic regression (MLR) was computed to build a first simple model using two classes: low (NYHA = 1 and NYHA = 2) and high (NYHA = 3) cardiovascular risk. Three assumptions for the regression analysis were checked [39]:

1. The absence of multicollinearity.
2. An outlier’s detection was performed by computing Cook’s distance and the Center Leverage Value adimensional coefficients.
3. According to Van Smeden et al. [40], the ratio between the sample size of the smallest class and the number of independent variables should be greater than 10.

### 2.4. Machine Learning: Tool and Algorithms

Several tools can be used to perform ML analyses: Tougui et al. performed a study on these tools in the context of heart disease classification [41] and identified Knime analytics platform as the best tool in terms of data manipulation, creating complex workflows, parameter tuning, and control of the algorithms. Moreover, this tool has already been used to perform biomedical studies also in fields such as ophthalmology and signal processing [42,43,44], and in cardiology [45,46].

The following three ML algorithms were considered to carry out the analysis.

k-Nearest Neighbors (kNN) is an instance-based statistical method. This algorithm is based on the hypothesis that records that are alike are likely to have properties that are alike. We can use this principle to classify data by placing it in the category with the most similar, or “nearest” neighbors. This method is based on the principle that the instances of a dataset will remain in close proximity with the other instances that have similar properties [47]. In thus method, a test example is classified by observing the class label of its adjacent neighbors. The KNN find outs the k-nearest instances to the query instance and identifies its class by finding the single most common class label [48].

ADA-B, short for Adaptive Boosting, is a ML meta-algorithm formulated by Yoav Freund and Robert Schapire [49]. It is adaptive in the sense that subsequent weak learners are tweaked in favor of those instances misclassified by previous classifiers. An ensemble of decision trees was considered to be the learner.

Naive Bayes (NB) algorithm is based on a simple application using Bayes’ theorem. Bayesian probability theory is rooted in the idea that the estimated likelihood of an event should be based on the evidence at hand. NB makes a “naive” assumption about the data, i.e., all the features in the dataset are equally important and independent. The Naive Bayes learner is trained by constructing a likelihood table. NB assumes class-conditional independence, which means that events are independent so long as they are conditioned on the same class value. The NB classifier greatly simplify learning by assuming that features are independent given classes. Although independence is generally a poor assumption, in practice NB often competes well with more sophisticated classifiers [50]. Despite its simplicity, the NB classifier has surprised ML researchers by exhibiting good performance on a variety of learning problems [51]. NB in fact has proven effective in many practical applications, including medical diagnosis [52,53] especially in the detection and prediction of heart diseases [54,55,56], as in the case under study.

## 3. Results

### 3.1. Statistical Analysis

#### 3.1.1. Univariate Statistical Analysis

The Kolmogorov Smirnov test for normality showed that only P, L and $\rho_{z}$ had a *p*-value greater than 0.05 indicating normality. Therefore, these underwent ANOVA test and, in the case of a significant result, also a Bonferroni post-hoc test; otherwise, the remaining variables underwent a Kruskal Wallis test and then, eventually, the non-parametric post-hoc test.

According to the results of the univariate statistical analysis (Table 2), 5 variables out of 9 obtained a statistically significant difference among the three groups of NYHA.

Among the 3D parameters, only $\rho_{y}$ achieved a statistical significance and was useful to distinguish both groups 1 and 2 from group 3, while $\rho_{x}$ was almost significant (*p*-value = 0.069). By contrast, N_p_ and D_p_ were both significant (*p*-values < 0.05) and could be used to distinguish respectively groups 1 and 3 and groups 2 and 3.

In summary, the post-hoc tests highlighted several differences between groups 1 and 3 or groups 2 and 3; no difference was found between groups 1 and 2.

#### 3.1.2. Multivariate Logistic Regression

In light of the results achieved in the univariate statistical analysis, the following analyses were performed distinguishing subjects in low (groups 1 and 2) and high (group 3) risk classes. The first group was made up of 138 patients while the other one was composed of 61 patients (where the 138 patients are sum of the 22 and 116 patients belonging to NYHA classes I and II cited in the “Study population” section).

To perform the MLR, the 3 assumptions (considered also in the “Statistical analysis” section) were evaluated [39]:

1. The multicollinearity was checked and Table S1 in the supplementary material shows the correlation among all the variables. At least one of the variables whose correlation was greater than 0.7 was removed from the model.
2. 8 outliers were removed (Figure S1 in the Supplementary Materials).
3. The ratio between the sample size of the smallest class and the number of independent variables was greater than 10 [40].
4. Table 3 shows the results obtained from the MLR.

HVE and *p* were not significant in the univariate statistical analysis, but they were considered in the multivariate analysis despite having only one significant result; differently, the strong significance of $\rho_{y}$ was confirmed also in this multivariate analysis (*p*-value < 0.001).

### 3.2. Machine-Learning Analysis

To classify patients into low and high-risk classes, it was carried out a ML analysis. First, a hold-out division intro train and test sets was performed, respectively 80% and 20% of the dataset. On the training set, a wrapper with a 10-fold cross-validation was applied to find the best subset of features maximizing the accuracy. The evaluation metrics were computed on the test set and the results are shown in Table 4.

ADA-B, kNN and NB achieved an accuracy greater than 80% and an Area Under the Receiver Operating Curve (AUCROC) greater than 0.70.

ADA-B and NB achieved satisfactory and similar results as regards both accuracy and AUCROC, but probably NB could be considered the best algorithm in this context considering the higher sensitivity (66.7% against 58.3%). KNN, instead, achieve the highest precision (83.3%) followed by ADA-B (77.8%).

## 4. Discussion

This study shows how ML algorithms can potentially help physiologists to correctly classify CHF patients considering the well-known NYHA severity scale using non-linear unconventional features. Moreover, the results of statistical analysis and ML confirm the potential benefit of these unconventional features to help clinicians in quantitative assessments.

First, the univariate statistical analysis showed the feasibility in distinguishing patients with low or high cardiovascular risk according to NYHA classification using features extracted through the Poincaré plot analysis. Then, the MLR helped to build a model with the above-mentioned features: HVE, P and $\rho_{y}$ were included and 2 out of 3 parameters were statistically significant in the model. Finally, a ML analysis was performed to classify patients into low or high cardiovascular risk, because no difference was found between NYHA class 1 and 2 in the univariate statistical analysis. This result has been found also in other works in the field [57], where several authors performed a discrimination between mild and severe heart failure.

In this research decision tree, random forests and multilayer perceptron were implemented without obtaining satisfactory results (data not shown for the sake of brevity). By contrast, the results of the ML analysis obtained from ADA-B, NB and KNN were shown because these were not highly affected by the unbalanced nature of the dataset. For this reason, while we had previously used an artificial augmentation of the dataset to perform the ML analysis [27], in this research we decided to show the analysis on the original dataset, without using any synthetic minority oversampling technique to balance the dataset with artificial data. Moreover, these three algorithms are based on different principles since ADA-B is an application of ensemble learning on the famous decision tree, KNN is an instance-based algorithm and NB is based on the a priori probability theorem of Bayes. All the presented algorithms achieved good results, but NB seemed to be the best one in terms of accuracy and AUCROC, although the sensitivity did not show high scores. The ML analysis (using the wrapper as features selection method) further confirmed the importance of L, N_p_ and $\rho_{y}$ that were identified twice as the best subset of features maximizing the accuracy.

A more detailed analysis based on the implementation of a greater number of ML algorithms coupled with the features selection by means of the wrapper methodology has showed more interesting results than our recent pilot paper [27]. Features reduction demonstrates an accuracy improvement both for the ADA-B algorithm (~8%) and the others. The investigation for appropriate subsets has greatly improved specificity scores (currently comprised between ~90% and 96%), while the algorithms confirm their weakness in sensitivity, confusing sometimes patients which do not belong to the group under examination. The most likely explanation of the negative result could be the distribution of the NYHA classifications in our dataset.

The combination of objective, methods and the selected Poincaré-related parameters expand the knowledge in this field setting this study apart from others. In fact, to our best knowledge, this is the first work which studies the possibility to distinguish CHF patients’ illness severity (considering NYHA classes) relying only on a subset of geometrical 2D and 3D parameters extracted from Poincaré plots. Similar works focused either on CHF patients and healthy subjects’ classification or performed ML classification considering also features related to the temporal and spatial domain.

Gonçalves and Oliveira [58] extracted several features from Poincaré plot which were previously subjected to a codification process. These features represented the input data for the implemented ML algorithms, namely multilayer perceptron and Support Vector Machines (SVM). The authors first evaluated the best number of cells and the best algorithms configurations to maximize the AUCROC and later showed satisfactory classification indices—specificity >90%, sensitivity >80% (100% for the multilayer perceptron) and accuracy ~95%—for both ML algorithms, highlighting the faster computation time for SVM. Similarly, Sepulveda-Suescun and co-workers [59] extracted from Poincaré plot 4 different features; 2 of these focused on 2 consecutive heart beats while the remaining 2 considered also data related to 5 consecutive heart beats, because the last ones demonstrated less sensible to irregular beats. The authors used the extracted features to differentiate short events of atrial fibrillation from normal sinus rhythm (in one case, considering only atrial fibrillation patients) by SVM showing classification performance up to ~98%. Rezaei and co-workers [60] finally presented similar results for heart arrhythmia classification. The authors extracted from Poincaré plot 16 parameters which were later statistically analyzed and used as input for a KNN algorithm. It was found a combination of 2 standard and 2 unconventional features was able to correctly classify (with performance far above 90%) cardiac signals related to different patients’ groups.

It could be started a discussion to compare the presented results citing previous studies whose aim was to distinguish CHF patients considering different severity classes for many cardiovascular diseases. These works consider several types of features extracted from time, spatial and non-linear—e.g., Poincaré plot conventional features—domains. The research of Tripoliti et al. summarizes multiple of these findings [57]. Nonetheless, to the authors’ best knowledge, no one of the previous works includes the 2D and 3D geometrical features (used in this work) for ML analyses. This evidence suggests an accurate comparison might request new investigations, where the presented geometrical features can be integrated with novel ones related to the previous mentioned domains. However, it might be useful a literature review and/or the design of preliminary studies to determine the reproducibility, reliability [25] and the prognostic value [34] of these features subsets. Anyway, readers can find several references which demonstrated the feasibility of CHF severity classification by ML algorithms using mixed features subsets in our pilot studies [26,27] and in [58].

In conclusion, the results obtained show NB, ADA-B and KNN (listed considering increasing sensitivities) can effectively classify CHF patients’ severity based on NYHA functional classification. Furthermore, this paper presents a novel application of specific and unconventional geometrical features extracted from Poincaré plot, previously investigated for CHF manual detection. Of course, other machine-learning workflows and statistical analyses could be performed and other conventional parameters or indexes correlating with CHF could be included to improve the evaluation metrics.

The main limitations of our work are linked to the features of the considered dataset, due to both clinical and technical considerations. On the one hand (i.e., from a technical point of view), the unbalanced distribution of patients assigned to the different NYHA classes in our dataset has—undoubtedly—negatively influenced ML scores, in particular sensitivity and specificity. Previous researchers have shown how unbalanced datasets can affect the results of machine-learning analyses and have suggested using both more than one single metric to represent the results and an AUCROC of the precision-recall diagram to quantitatively evaluate the reliability of the models [61]. In this regard, a bigger and more balanced dataset would be useful in the future to improve the results that can be obtained by applying ML on the features extracted through Poincaré plot analysis, making this methodology more robust and reliable. Indeed, augmenting the dataset would allow future researchers to test also other powerful algorithms which we had not the chance to use in this research due to the unbalanced dataset. On the other hand (i.e., from a clinical point of view), the considered dataset lacked enough NYHA class IV patients and overlooked the potential separation of NYHA class II patients in the subclasses NYHA IIS and IIM (where the indexes “S” and “M” indicate a slight or moderate limitation of physical activity, respectively) [62]. Clearly, further research using such accurate dataset would be desirable to understand further potentialities of the proposed methodology.

Finally, the implemented workflow of research may be affected by the manual subjective revision of all the single beats before building the Poincaré map; a future development could consist of building an automatic process also for this step of the research.

## Figures and Tables

**Table 1:** Clinical characteristics of the population studied.

Age	Male	Cause [%]			LVEF	VPC	NSVT
[years]	[%]	Ischemic	Idiopathic	Other	%	n/h	%
54	87	50	45	3	23	13	37

Abbreviations: LVEF: left ventricular ejection fraction; VPC: ventricular premature contractions, NSVT: non-sustained ventricular tachycardia.

**Table 2:** Univariate statistical analysis performed through one-way ANOVA/Kruskal Wallis plus post-hoc tests for all the variables. Post-hoc analyses were reported only for significant tests.

Variables	NYHA = 1	NYHA = 2	NYHA = 3	*p*-Value	Post-Hoc Classes *p*-Value
L	631.14 ± 173.27	594.74 ± 174.79	512.05 ± 188.62	0.005 ‘	1–3, 0.024 2–3, 0.012
HVE	257.02 ± 96.37	235.74 ± 95.25	229.09 ±82.00	0.518 ^	NA
A	13,194.09 ± 7313.36	14,094.22 ± 10,389.57	10,975.41 ± 8714.27	0.040 ^	2–3, 0.041
*p*	61.72 ± 12.43	55.99 ± 14.39	56.70±13.49	0.210 ‘	NA
$N_{p}$	36.82 ± 29.40	27.79 ± 17.69	22.90 ± 18.30	0.010 ^	1–3, 0.019
$D_{p}$	48.04 ± 9.60	53.89 ± 19.90	61.27 ± 22.10	0.025 ^	2–3, 0.047
$\rho_{x}$	120.22 ± 32.81	113.51 ± 28.00	105.33 ± 23.25	0.069 ^	NA
$\rho_{y}$	113.62 ± 39.06	105.54 ± 39.62	84.55 ± 37.49	0.001 ^	2–3, 0.002 1–3, 0.007
$\rho_{z}$	798.46 ± 134.32	811.17 ± 118.53	773.51 ± 149.45	0.195 ‘	NA

‘ = ANOVA. ^ = Kruskal Wallis. NA = Not Applicable.

**Table 3:** The results—odds ratio with 95% confidence interval (CI) and *p*-values—of the multivariate logistic regression. Accuracy of MLR = 78.4%.

Variables	Odds Ratio (95% CI)	*p*-Value
L	NI	NI
HVE	0.997 (0.993–1.002)	0.229
A	NI	NI
*p*	0.983 (0.966–1.000)	0.046
$N_{p}$	NI	NI
$D_{p}$	NI	NI
$\rho_{x}$	NI	NI
$\rho_{y}$	1.027 (1.015–1.040)	0.000
$\rho_{z}$	NI	NI

NI = Not included.

**Table 4:** Evaluation metrics and features included per each algorithm.

Algorithms	Accuracy [%]	Sensitivity [%]	Specificity [%]	Precision [%]	AUCROC [%]	Features Selected
ADA-B	82.5	58.3	92.9	77.8	0.756	L, P
KNN	80.0	41.7	96.4	83.3	0.702	$L,\;N_{p}$ $,\;\rho_{y}$
NB	82.5	66.7	89.3	72.7	0.747	$L,\;N_{p}$ $,\;\rho_{y}$

Abbreviations: ADA-B: Ada Boost; KNN: K-Nearest neighbors; NB: Naive Bayes.

## Data Availability

The datasets generated and/or analyzed during the current study are not publicly available due to privacy policy but are available from the corresponding author on reasonable request.

## Supplementary Materials

The following are available online at https://www.mdpi.com/article/10.3390/bioengineering8100138/s1, Table S1: Bivariate Pearson’s correlation among all the independent variables, Figure S1: Outliers were found through the Cook’s distance vs. Center Leverage Value graph.
