# Supplementary material for: Extracting Features from Poincaré Plots to Distinguish Congestive Heart Failure Patients According to NYHA Classes
Source: Bioengineering (Basel). 2021 Oct 3;8(10):138. doi: 10.3390/bioengineering8100138 (PMC8533203; doi:10.3390/bioengineering8100138)
Supplement: Supplementary file 1 [file bioengineering-08-00138-s001.zip › bioengineering-1358016-supplementary.pdf]

Table S1. Bivariate Pearson's correlation among all the independent variables

		L	HVE	A	P	Np	Dp	Px	Py	Pz
L	Pearson's Correlation	1	,477**	,488**	,273**	,569**	-,588**	**,798****	**,882****	,598**
	Sign. (p-value)		,000	,000	,000	,000	,000	,000	,000	,000
	N	199	199	199	199	198	198	198	198	198
HVE	Pearson's Correlation	,477**	1	,377**	,025	,362**	-,300**	,396**	,428**	,198**
	Sign. (p-value)	,000		,000	,722	,000	,000	,000	,000	,005
	N	199	199	199	199	198	198	198	198	198
A	Pearson's Correlation	,488**	,377**	1	,191**	,357**	,169*	,614**	**,751****	,309**
	Sign. (p-value)	,000	,000		,007	,000	,017	,000	,000	,000
	N	199	199	199	199	198	198	198	198	198
P	Pearson's Correlation	,273**	,025	,191**	1	,148*	-,020	,346**	,296**	,216**
	Sign. (p-value)	,000	,722	,007		,037	,774	,000	,000	,002
	N	199	199	199	199	198	198	198	198	198
Np	Pearson's Correlation	,569**	,362**	,357**	,148*	1	-,451**	,441**	,553**	,570**
	Sign. (p-value)	,000	,000	,000	,037		,000	,000	,000	,000
	N	198	198	198	198	198	198	198	198	198
Dp	Pearson's Correlation	-,588**	-,300**	,169*	-,020	-,451**	1	-,157*	-,337**	-,467**
	Sign. (p-value)	,000	,000	,017	,774	,000		,027	,000	,000
	N	198	198	198	198	198	198	198	198	198
Px	Pearson's Correlation	,798**	,396**	,614**	,346**	,441**	-,157*	1	**,889****	,354**
	Sign. (p-value)	,000	,000	,000	,000	,000	,027		,000	,000
	N	198	198	198	198	198	198	198	198	198
Py	Pearson's Correlation	,882**	,428**	,751**	,296**	,553**	-,337**	,889**	1	,560**
	Sign. (p-value)	,000	,000	,000	,000	,000	,000	,000		,000
	N	198	198	198	198	198	198	198	198	198
Pz	Pearson's Correlation	,598**	,198**	,309**	,216**	,570**	-,467**	,354**	,560**	1
	Sign. (p-value)	,000	,005	,000	,002	,000	,000	,000	,000	
	N	198	198	198	198	198	198	198	198	198

**. Significant at 0,01.

*. Significant at 0,05.

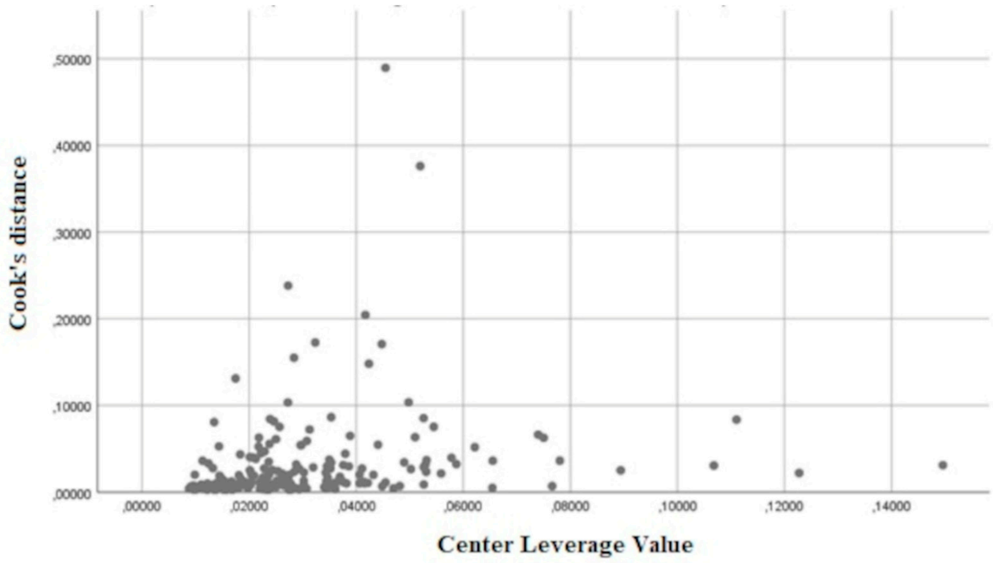

Figure S1. Outliers were found through the Cook's distance Vs Center Leverage Value graph
